# Young patients', parents', and survivors' communication preferences in paediatric oncology: Results of online focus groups

**DOI:** 10.1186/1471-2431-7-35

**Published:** 2007-11-09

**Authors:** Marieke Zwaanswijk, Kiek Tates, Sandra van Dulmen, Peter M Hoogerbrugge, Willem A Kamps, Jozien M Bensing

**Affiliations:** 1 NIVEL, Netherlands Institute for Health Services Research, P.O. Box 1568, 3500 BN Utrecht, The Netherlands; 2 Department of Paediatric Hemato-Oncology, University Medical Centre St. Radboud, Nijmegen, The Netherlands; 3 Department of Paediatric Oncology, University Medical Centre Groningen, The Netherlands; 4 Department of Clinical and Health Psychology, Faculty of Social Sciences, Utrecht University, The Netherlands

## Abstract

**Background:**

Guidelines in paediatric oncology encourage health care providers to share relevant information with young patients and parents to enable their active participation in decision making. It is not clear to what extent this mirrors patients' and parents' preferences. This study investigated communication preferences of childhood cancer patients, parents, and survivors of childhood cancer.

**Methods:**

Communication preferences were examined by means of online focus groups. Seven patients (aged 8–17), 11 parents, and 18 survivors (aged 8–17 at diagnosis) participated. Recruitment took place by consecutive inclusion in two Dutch university oncological wards. Questions concerned preferences regarding interpersonal relationships, information exchange and participation in decision making.

**Results:**

Participants expressed detailed and multi-faceted views regarding their needs and preferences in communication in paediatric oncology. They agreed on the importance of several interpersonal and informational aspects of communication, such as honesty, support, and the need to be fully informed. Participants generally preferred a collaborative role in medical decision making. Differences in views were found regarding the desirability of the patient's presence during consultations. Patients differed in their satisfaction with their parents' role as managers of the communication.

**Conclusion:**

Young patients' preferences mainly concur with current guidelines of providing them with medical information and enabling their participation in medical decision making. Still, some variation in preferences was found, which faces health care providers with the task of balancing between the sometimes conflicting preferences of young cancer patients and their parents.

## Background

Good communication in health care is generally considered to consist of three broad tasks [1]. The first, interpersonal relationship building, is a result of mutual respect, trust and empathy. It is thought of as prerequisite for the other two elements of good communication: information exchange and participation in the decision-making process to the degree that is desired and feasible [2].

Characteristics of communication in paediatric oncology complicate the execution of these tasks. First, open communication about the illness is regarded as the best policy for child and parents [3], as it leads to an improved knowledge and understanding of the illness, and decreases anxiety and depression [4]. Information provided in paediatric oncology, however, is generally complex and emotionally charged in nature, and usually involves a degree of uncertainty [5].

Secondly, paediatric oncology care involves a wide array of health care professionals and care settings, which requires sufficient collaboration and communication within and between these various care settings. These characteristics entail the risk of miscommunication and misunderstanding, which, in turn, may negatively affect trust in health care professionals [6-8] and participation in the decision-making process [9]. Characteristics surrounding the diagnosis of childhood cancer, such as the urgency in taking action and the threat of death may also cause parents and patients to feel that choices are limited, and that they have to rely on health care providers to make treatment decisions [9,10].

Paediatric oncology care entails at least a triad, involving the medical care team, patient and parents [11]. It is increasingly being acknowledged that children should be involved in decisions about health care [12-16]. Recently developed guidelines encourage health care providers to share developmentally relevant medical information with the child to enable the child's active participation in the decision-making process [17,18]. However, observations as well as self-reports show that young people's participation in consultations is often limited [19-23]. This may be a result of children's own choice, but it may also be caused by adults' protectiveness or incomplete knowledge of children's competence to understand medical information and to be an active participant in the medical consultation [cf. [13,15]]. Some children have reported to be dissatisfied with their non-participant status, which can hamper their ability to make sense of their illness and to have their interests taken into account [19,20,23]. Remarkably little is known about the preferences of young patients and parents involved in communication in paediatric oncology. Moreover, parents have usually been included in studies as source of information about their children. As Dixon-Woods et al. [24] argue, this has had a doubly silencing effect, as a result of which the unique perspectives of neither parents nor children are considered.

Perceptions of what constitutes good communication in terms of interpersonal relationships, information exchange and participation in the decision-making process may differ both between and within groups of child patients and parents [c.f. [9,23,25-29]]. Insight into the needs and preferences of young cancer patients and their parents may contribute to successful communication, and thereby positively affect patients' and parents' satisfaction with communication.

Focus group discussions are a means to explore respondents' needs and preferences. These discussions have specifically been recommended in research with children, as they allow them to use their own words in formulating responses, and provide them the opportunity to resist researchers' control of the research process [30]. The Internet is increasingly being used as a medium for focus groups, and the feasibility and effectiveness of online focus group discussions have been reported extensively [31-34]. Online focus groups have several advantages compared to traditional face-to-face focus groups [32-37], for both participants and researchers. Firstly, this method allows spatiotemporally separated participants to join the discussion from their home and at a convenient time, which is particularly important in case of severely ill children. The higher level of anonymity in online discussions has also been shown to allow participants to speak more freely and provide more honest answers, particularly regarding sensitive topics. Thirdly, the written contributions of participants yield immediately available data, which considerably decreases costs and time needed for data entry and analysis. Children's familiarity with the Internet further pleads in favour of this new methodology.

The aim of this study is to gain insight into the interpersonal, informational, and decisional preferences of participants involved in paediatric oncology, using online focus group discussions. Three groups of participants are involved: (a) child and adolescent patients (aged 8 to 17 years) currently in active treatment for childhood cancer, (b) parents of these patients, and (c) children and adolescents (aged 8 to 17 years at diagnosis) who have been successfully treated for childhood cancer in the preceding five years.

## Methods

### Participants

Communication needs and preferences were examined by means of online focus groups. Three groups of eligible participants were selected by consecutive inclusion in two Dutch university oncological wards, and asked to participate in separate focus groups (Table 1). The first group (referred to as 'patients') consisted of children and adolescents (8 to 17 years old), who had been diagnosed with childhood cancer 6 weeks to 1 year ago, and who were currently in active treatment. Separate focus groups were organised for children (aged 8 to 11 years) and adolescents (aged 12 to 17 years). Parents of patients were asked to participate in a separate focus group. The third group (survivors) consisted of children and adolescents who had been 8 to 17 years old when diagnosed with childhood cancer, and whose treatment had been successfully finished during the preceding five years. Insufficient mastery of the Dutch language, a lag in development, treatment for secondary tumours, and being in a palliative phase of care (oncologists' evaluations) were used as exclusion criteria.

**Table 1:** Participants of online focus groups

	Patients			Parents			Survivors		
	W1	W2	Total	W1	W2	Total	W1	W2	Total

Approached: N	7	24	31	14	48	62	30	26	56
Agreed: % (N)	57.1 (4)	29.2 (7)	35.5 (11)	42.9 (6)	25.0 (12)	29.0 (18)	36.7 (11)	30.8 (8)	33.9 (19)
Participated: % (N)	28.6 (2)	20.8 (5)	22.6 (7)	21.4 (3)	16.7 (8)	17.7 (11)	33.3 (10)	30.8 (8)	32.1 (18)

Note: W1 = ward 1; W2 = ward 2

For practical reasons, the recruitment of patients and parents was carried out differently in the two oncological wards. In the first ward, 7 families (7 patients and 14 parents) were informed about the study objectives and methods and asked to participate by a nurse practitioner, when they visited the ward in the period between October and December 2005. Twenty-four eligible families (24 patients and 48 parents) in the second ward were selected from an electronic patient recording system, based on their order of appearance on the ward. They were informed about the study and asked to participate by a letter on behalf of the head of the Department of Paediatric Hemato-Oncology in November 2005, and received a reminder two weeks after the initial letter.

Family members were able to individually chose to participate, meaning that not necessarily both parents and the child of the participating families were included in the study. Of the 31 families that were approached in total, written consent was obtained from 13 families (11 patients and 18 parents). Eight of these families (7 patients and 11 parents) actually participated. Responding (N = 7) and non-responding (N = 24) patients were comparable with respect to current age, age at the time of diagnosis and gender. Responding (N = 11) and non-responding (N = 51) parents were comparable with respect to gender (the age of non-responding parents was not available). In two of the families that did not participate despite their initial consent, the child was severely ill at the start of the online focus group. For the remaining three families, reasons for not participating despite their initial consent were not known.

Because survivors did not visit the oncological ward on a regular basis, eligible participants for the survivors group were selected from electronic patient recording systems in both wards. A total number of 56 survivors were first informed about the study by mail from the hospital in February 2006, and, if necessary, received a reminder two weeks later. Written consent was obtained from 19 of them, and 18 actually participated. Responding (N = 18) and non-responding (N = 38) survivors were comparable with respect to current age, age at the time of diagnosis and gender.

Characteristics of participating patients, parents and survivors are reported in Table 2. Research ethics approval was obtained for the participating medical centres (METC 2005-050 and AMO 05/074).

**Table 2:** Characteristics of participants of online focus groups

	Patients N = 7	Parents N = 11	Survivors N = 18
Age: mean (range)	11.6 (8–16)	45.9 (37–72)	15.5 (10–19)
Age at diagnosis: mean (range)	10.4 (8–15)	-	11.6 (8–16)
Male gender: % (N)	42.9% (3)	45.5% (5)	38.9% (7)
Diagnosis: % (N)			
Leukaemia	42.9% (3)	-	55.6% (10)
Brain tumour	28.6% (2)	-	11.1% (2)
Lymphoma	14.3% (1)	-	16.7% (3)
Bone tumour	-	-	16.7% (3)
Soft tissue sarcoma	14.3% (1)	-	-

### Procedure

The online focus groups were conducted in an asynchronous form [31,32,36], i.e. participants could read others' comments and could respond at any time, not necessarily when anyone else was participating. This allowed participants to respond from their home and at any time convenient to them. The participants received individual login names and passwords, with which they could anonymously access the Internet focus group website during one week. To ensure anonymity, participants were asked not to mention their own names or addresses, or the names of health care providers. On the third day, participants who had not yet reacted received an email to invite them to respond.

A new question was introduced by the researchers on each of the first five days. As recommended in previous focus group research [38], we started with (1) a concrete question concerning participants' experiences with the diagnostic consultation, before turning to more general and abstract issues, such as (2) role delineation between parent and young patient with respect to information exchange, (3) preferences concerning participation in decision making, (4) role delineation between physicians and nurses in communication, and (5) role delineation between parent and patient with respect to care when the child is at home. To give an impression of the kind of questions that were used in the focus groups, questions for young children are reported in Table 3. Questions for the other groups of participants were comparable in content, but the wording was adapted to the age range of the participants.

**Table 3:** Questions used in young patients' focus group

Day 1	Please think back to the consultation in which you were first informed about your illness and treatment. Who told you this? Who were with you when you heard the news? What did you like about that conversation? About which aspects were you less satisfied? What would you've rather done differently?
Day 2	There is a law, which states that ill children should know exactly what's wrong with them and what could be done about it. We'd like to know your opinion about this. Do you like to talk to doctors yourself or do you prefer your parents to do the talking? You may also don't like to be present at all during important consultations, but rather hear everything from your parents afterwards.
Day 3	The law also says that your parents and the doctor will decide what's best for you until you're 12 years old. Do you think that children are also able to make decisions about their treatment or about the way in which certain examinations should be performed? Can you give an example of things you do or you don't want to make decisions about?
Day 4	You've probably talked to quite a few doctors and nurses since you've been ill. Does it matter who you talk to? Which things do you prefer to discuss with your doctor? Which things would you rather discuss with a nurse? If you'd have to explain to doctors and nurses what they should keep in mind when talking with children about their illness or treatment, what would you suggest?
Day 5	Even when you're very ill, you don't have to be in the hospital all the time. Doctors think it's very important for ill children to be at home and go to school as much as possible. That may be difficult sometimes, because you and your parents have to keep in mind many things, such as your medicines, food, and things you can and cannot do. We'd like to know how you handle these things. Do you have to think about these things yourself or do you leave those things to your parents?

On the sixth and seventh day, participants were invited to introduce new issues they considered relevant in communication in paediatric oncology. Questions of the previous days remained open for responses during the whole week. The researchers acted as moderators by regularly checking the postings, and by asking additional questions to clarify participants' views if necessary.

Characteristics of the reactions to the topics raised during the first 5 days of the focus groups are reported in Table 4. Young child patients tended to direct their comments to the moderators rather than to each other, whereas adolescent patients and survivors developed a more interactive way of responding by reacting actively to each others' contributions. Parents entered long and well-considered postings at varying times of the day.

**Table 4:** Characteristics of response in the online focus groups

	Patients N = 7	Parents N = 11	Survivors N = 18
Total number of postings	47	46	111
Postings per day*	9 (9–11)	9 (4–12)	22 (20–25)
Postings per participant*	7 (4–11)	4 (1–8)	6 (1–15)
Number of main topics covered per participant*	5 (4–5)	3 (1–5)	4 (1–5)

Note. Results are reported for the first 5 days of the online focus groups, with 5 main topics. * Reported are means and ranges

Topics were derived from the literature and were the same for the three groups of participants. Issues that were emphasized differed between groups, because participants were invited to react on each others' contributions. Needs and preferences that were expressed by the focus group participants are listed in Table 5. In describing the needs and preferences of focus group participants (see the Results section), any differences between or within the three groups of participants are explicitly mentioned. The term 'participants' will be used to indicate all groups whenever the three groups had similar views about certain aspects of communication.

**Table 5:** Needs and preferences expressed in focus groups

		Patients (N = 7)	Parents (N = 11)	Survivors (N = 18)
**Interpersonal relationships**	Honesty	x	x	x
	Reassurance, support and empathy	x	x	x
	Being taken seriously		x	x
	Sufficient time for communication	x	x	x
	Time to come to terms with upsetting information	x	x	x
	Trust in health care professionals' expertise		x	x
	Not being constantly addressed as patient	x	x	x
	Acquaintance with child patient	x	x	x
**Information exchange**	Being fully informed	x	x	x
	Clarity of information	x	x	x
	Opportunity to ask questions during the consultation	x	x	x
	Avoidance of technical jargon	x	x	x
	Additional written information		x	x
	Unambiguous information		x	
	Differentiation in amount and kind of information		x	x
	General information at diagnosis, detailed information later	x	x	x
	Repetition of important information		x	x
	Written summary of consultations		x	
	Notification of the timing of consultations	x	x	x
	Presence of patient during consultations	x	x	x
	Parent-patient role delineation in information exchange	x	x	x
	Accessibility of health care providers	x	x	x
**Participation in decision making**	Level of participation in major decisions	x	x	x
	Level of participation in minor decisions	x	x	x

Note. x = need or preference is expressed in focus group

Key aspects of participants' views on communication in paediatric oncology were selected. Two authors (MZ and KT) each read the transcripts independently and constructed a preliminary thematic coding scheme. Disagreements during this process were discussed until consensus was achieved.

## Results

### Preferences concerning interpersonal relationships

An open and honest communication was valued highly by the participants, as is shown in the following citation: *'Physicians and nurses should be honest about what's going to happen, because if they fool me once I'll never believe them again.' *(patient, aged 8). The participants realised, however, that being fully and truthfully informed about the illness and treatment could be confronting at first. Patients and survivors emphasized that openness in communication applied not only to health care providers, but that they themselves should provide honest and open information about their physical well-being as well. Parents expected health care providers also to be honest about not knowing certain things.

All participant groups expressed a need for reassurance, support, and empathy from physicians and nurses. Parents also wanted to be taken seriously. This included being addressed to as an adult, being informed about the reasons for certain actions concerning their child, and acknowledgement of their role as parents and experts about their child. Survivors expressed another aspect of being taken seriously: *'Nurses shouldn't ask every day: "How are you?" When you've heard that question thirty times already, it's very tiring to have to explain for the thirty-first time that you're feeling very bad' *(survivor, aged 19).

The participants wanted physicians and nurses to take sufficient time to talk, not merely to actually listen to their views, and adequately and calmly explain aspects of the child's illness and treatment, but also to be allowed some time to come to terms with upsetting information. Because of the latter aspect, diagnostic consultations were reported to be often split up in two parts, between which parents and patients were given some time alone to be able to come to terms with the shock of the diagnosis.

Trust in health care professionals' expertise was mentioned by parents and survivors as another important aspect of interpersonal communication, associated with their limited knowledge of paediatric oncology (see also the section concerning decision-making preferences): '*With proper assistance, explanation, and information, a sense of trust will develop, on the basis of which we assume that physicians will be able to make the best considerations and may be able to explain their choices' *(parent, aged 45).

The participants reported that it made no difference with which health care provider they discussed the child's illness and treatment, as long as their questions and concerns were adequately addressed. In practice, contacts with nurses were reported to be more informal than discussions with physicians. Nurses talked about patients' day-to-day activities and hobbies, made fun with patients, and listened to their daily concerns. This kind of communication, which did not constantly address them as patients, was highly appreciated by patients and survivors. Discussions with physicians were more directly related to illness and treatment. *'I discuss how I'm doing and my future life and so on with my physician, but I guess that's mainly because he has to write it down. I also talk about these things with nurses, but they seem much more interested. So, a hint for physicians: make sure it does not only look like you have to write everything down, but really listen to and talk with the child' *(survivor, aged 17).

All participants wanted the physician to be well acquainted with the child's individual situation, and therefore preferred continuity of care, i.e. they preferred consultations to be with the same physician during the entire course of treatment. Parents and survivors expressed a clear dislike of repeatedly having to explain the child's situation, and felt that the variation in physicians could prevent the detection of any changes in the child's condition. They also preferred to be in contact with only one physician because this provided the possibility of establishing a trusting relationship, created unity in terms being used, and prevented potential miscommunication between physicians.

### Preferences concerning information exchange

The participants stressed young patients' basic right to be fully informed about the illness and treatment. A young patient (aged 10) expressed an interesting view in this respect: *'I always listen in, even when I have to do something else. (...) I usually want to know everything they talk about.' *Participants also acknowledged, however, that patients differ in the amount and kind of information they prefer to receive. Particularly information about survival rates and prognosis were mentioned as topics that not all child cancer patients want to be informed about.

The importance of clarity of information about illness and treatment was emphasized by all participants, who also wanted to be given the opportunity to ask questions to increase clarity. Most participants wanted technical jargon to be avoided, although one parent preferred physicians to use and explain medical jargon, because that way he could search for and understand additional information in books or on the Internet. Some parents preferred to receive written information about the illness or wanted to be informed where to find additional information. Parents indicated that it is important to prevent the provision of contradictory information by different health care providers.

Parents and survivors expressed their wish to adapt the content of information to the age and cognitive capacities of the patient. Adolescent survivors emphasized the lack of information designed specifically for their age. They preferred information to be adapted to their specific needs as adolescents, instead of being addressed to as either children or adults.

The shock of being informed about the diagnosis prevented the participants from adequately absorbing all relevant information. They therefore preferred to receive only general information at the time of diagnosis, followed by more detailed information during subsequent consultations. Survivors also acknowledged the dilemma with which physicians are faced, with the requirement to fully inform young patients and parents about illness and treatment, and on the other hand the awareness that the majority of this information is lost in the shock of hearing the diagnosis.

Parents and survivors mentioned the importance of repeating information about the illness and treatment. Survivors particularly emphasized that patients should not feel embarrassed to ask the same questions again if this was necessary to completely understand the information. Parents mentioned the usefulness of receiving a written summary of consultations, which made it possible to reread important information.

Participants preferred to be notified in time when consultations were going to take place, but they reported different reasons for this preference. Parents wanted to be able to prepare themselves and to make sure that both parents could be present, particularly during the diagnostic consultation. Child patients and survivors preferred to know the timing of consultations for more practical reasons, such as not having to get up too early.

Preferences regarding the presence of young patients during consultations varied considerably. Whereas some patients and survivors wished to be present during all consultations, others indicated that they did not mind their parents occasionally speaking privately with the physician, and that they preferred to be informed by their parents instead of by the physician. Patients' absence during consultations could, however, give them the impression that important information about their illness was held back.

Parents' reports varied from never to sometimes having had consultations without their child being present. Parents who chose to discuss things with the physician in the absence of their child and thereby functioned as intermediate in the information exchange between physician and patient, mostly did this because they wanted to shield their child from potentially upsetting information, or because they considered their child too young to be burdened with such information. Their decision concerning the child's presence was also influenced by the cognitive abilities, preferences, and emotional and physical condition of the child. Parents who preferred their child to be informed directly by the physician indicated that they considered the physician better qualified to clearly explain aspects of the child's illness and treatment, to answer the child's questions, and to prevent any misunderstandings, without getting too emotionally involved. Some parents experienced their child's absence or presence during consultations not as a result of their own decision making, but rather as a consequence of convention.

Survivors emphasized that young patients should be explicitly involved in deciding how they should be informed and which information they should receive. This may also bring about some difficulties, as a survivor (aged 19) stated: *'I don't think asking a child to indicate what he or she wants or doesn't want to know will work out right. There may be a lot of information that you would like to know, but you may not even know that it exists. Particularly at the start. You shouldn't make a child think too much at that time, because he or she is thinking of other things then.'*

Patients sometimes preferred to use their parents to facilitate communication with health care providers: *'When my parents notice that I don't know the answer, they help me. They also help me when I forget to ask or say something. (...) I sometimes prefer my parents to do the talking because I sometimes don't know what to say' *(patient, aged 15). *'(...) if you're having a hard time, it's useful that your parents also hear what's being said, so that they can tell you everything once more' *(patient, aged 16). Despite this occasional use of their parents as facilitators of the communication, patients and survivors preferred to be the ones to whom information and questions were addressed.

The participants generally knew who to turn to with questions regarding illness or treatment. Parents and survivors stressed the importance of being able to reach health care providers at all times, although their opinions about the actual accessibility of health care providers varied. Particularly in the course of treatment and when the child was at home in periods between treatments, parents and survivors reported having difficulty in reaching health care professionals to answer their questions. Some of them suggested the introduction of an e-mail service, which would facilitate the discussion with health care providers, particularly concerning sensitive topics.

### Preferences concerning participation in the decision-making process

The majority of participants preferred decisions about treatment to be made in collaboration between patients, parents, and health care providers. This preference concerned major decisions about the execution of treatments as well as decisions concerning procedures surrounding treatment and examinations, such as the timing of appointments and the use of sedatives. Only one survivor (aged 11) and two patients (both aged 10) expressed a preference for a passive role in making major decisions on treatment. The two patients, however, did want to take part in less important decisions. Although parents could be of assistance in reaching a decision, and, in doing so, could affect the decision-making process, survivors and adolescent patients emphasized that they should be the ones to make the final decision. Some survivors referred to the importance of patient age in determining the appropriate level of patients' participation in the decision-making process: *'(...) if you're older than fifteen, you're allowed to have a say in the decision and to decide for yourself sometimes. If you're younger than fifteen, you should decide together. I think children younger than fifteen don't really know what's good and bad for them' *(survivor, aged 17).

Despite their general preference for collaborative decision making, participants indicated that characteristics of the situation sometimes prevented them from being actively involved in deciding about treatment. In some cases they felt they did not have a choice, as the patient's only chance of getting better was to be treated, and there was a prescribed treatment protocol. Other reasons mentioned as preventing participation in decision making were: lack of sufficient knowledge of the illness and treatment or trust in the physician's expertise, practical circumstances, and the patient being too ill or depressed to decide.

## Discussion

To our knowledge, this study is the first to investigate communication needs and preferences in paediatric oncology from the perspectives of child cancer patients, parents, and survivors of paediatric cancer. The focus group participants expressed detailed and multi-faceted views regarding their needs and preferences. The simultaneous inclusion of the three groups enabled comparison of their views, which revealed similarities and differences between and within groups.

There was unanimity among participants with respect to the majority of interpersonal aspects preferred in communication. In line with previous research [19], the participants valued honesty, reassurance and support from health care providers, sufficient time for communication, and continuity of health care providers. A subtle difference between parents and survivors was found in the definition of the need to be taken seriously. The only aspect of communication valued highly by patients and survivors, but not mentioned by parents, was communication about non-medical issues, which gave patients and survivors the impression of not being constantly addressed as patients.

Young patients, parents, and survivors also agreed on the importance of several aspects of information exchange, such as the need to be fully informed and to have the opportunity to ask questions, and the accessibility of health care providers. Previous research showed that patients and parents prefer to receive illness-related information in face-to-face situations. When health care providers' workloads limit the possibility of being informed face-to-face, patients and parents prefer to receive information in writing or through the Internet [39,40].

Considerable differences in views within the groups of participants were found for the preference of the patient's presence during consultations. Mack et al. [41] reported a positive association between the presence of the child patient during consultations and the patient's age, which was reflected in parents' contributions in the present study. Patients' and survivors' preferences concerning their presence during consultations did not show a clear association with age, however.

The absence of the child patient during consultations directly affects the role delineation between parents and child in information exchange. It causes the parents to be managers of what their child is told about the illness, and when and how this information is provided [23]. Young patients differed in the extent to which they were satisfied with the parental role in communication. Some thought communication to be constrained by their parents, whereas others explicitly used their parents in the communication with health care providers. In the latter case, parents performed the roles reported by Young et al. [23]: they functioned as facilitators of communication, communication buffers (i.e. patients use their parents to answer difficult questions), or communication brokers by repeating or clarifying important information.

In line with previous research [9,19,29], major decisions about the execution of treatments as well as decisions concerning procedures surrounding treatment and examinations were generally preferred to be taken in collaboration between health care providers, parents, and young patients. Although the number of participants who preferred passive involvement in treatment decision making was small, the variation in preferences highlights the need to consider individual differences.

Survivors and adolescent patients emphasized that parents could be of assistance in reaching a decision and thereby affect the decision-making process, but that they themselves should be the ones to make the final decision. This statement provides confirmation for the distinction between decisional priority and decisional authority made by Whitney et al. [42]. The person who has decisional priority takes the lead in the process of choosing between possible treatment options, resulting in a recommendation or request which prepares the ground for the final decision. The actual decision to accept or reject the proposed option is made by the person who has decisional authority. Although parents may have decisional priority, survivors and adolescent patients think that they should have decisional authority, which is in line with the Dutch medical treatment act.

According to Whitney et al. [42], the person who assumes decisional priority in paediatric oncology depends on the number of available options and the curability of the specific type of cancer. When there is, for instance, one clear best treatment option and cure is probable, decisional priority lies primarily in the physician's hands. This corresponds with participants' reports of not always being actively involved in deciding about treatment, despite their preference for collaborative decision making. In situations where one clear best treatment option is lacking, patients and parents have a more active role in the decision-making process [42]. This is in line with the concept of professional 'equipoise', which is believed to enable collaborative decision making [43].

### Methodological reflections

Online focus groups have mainly been used in adult populations and to our knowledge, these groups have not been previously used for children in paediatric settings. In general, the online focus group methodology proved to be a feasible tool for collecting data from hard-to-include respondents, such as children in active treatment for childhood cancer and their parents. Young patients, their parents, as well as survivors could be actively engaged over a one-week period. They provided elaborate and detailed responses to the questions posed on the focus group website. The fact that not merely the parents, but also the children and adolescents in our study were able to clearly articulate their needs and preferences, argues for the use of this method in future research to further reveal the previously often neglected opinions of children.

For practical reasons, recruitment of patients and parents took place in different ways in the two oncological wards. In the first ward, eligible participants were approached in person, whereas participants were contacted by mail in the second ward. This resulted in different response rates (Table 1). Although recruitment by mail provides the possibility of reaching a larger sample in a shorter amount of time, contacting eligible participants in person resulted in higher rates of agreement to participate. However, the percentages of respondents who did not participate despite their initial consent were also higher in the first ward. Eligible participants who are approached in person may find it harder to refuse participation, leading to higher rates of agreement to participate, but probably also to higher rates of secondary attrition. Future studies are needed to further investigate the best way of recruitment in comparable samples.

By recruiting respondents through hospitals instead of relying on self-selection through the Internet, we were able to minimize the selection bias frequently mentioned as drawback of online research [44]. Still, children and parents with stronger preferences for participating in medical communication and decision making may have been more likely to participate in the study, thereby overestimating the preference for participation. The aim of this qualitative study was, however, not to generalize the results, but rather to increase our understanding of processes that were hardly studied before. Focus groups are typically meant to elicit data on the views and opinions of small groups of people. Emphasis is placed upon achieving a depth of understanding instead of upon generalization of the findings [45].

The results of this study may be regarded as a first exploratory glance at the needs and preferences of patients and parents involved in communication in paediatric oncology care. To be able to adapt communication in paediatric oncology to the preferences of young patients and parents, more insight into these preferences is needed. This involves not only the validation of the current results in larger samples, but also studying associations between sociodemographic, illness, or treatment related variables and participants' preferences in communication, and studying the changes in preferences that may occur during the course of the illness [46,47]. These subjects will be addressed in a study currently being conducted by this research team.

## Conclusion

Current guidelines of sharing developmentally relevant medical information with young patients to enable their active participation in decisions about their own health care mainly concur with children's and adolescents' preferences. The majority of young patients and survivors wished to be fully and truthfully informed and preferred to participate in treatment decision making. Still, some variation in preferences was found, which faces health care providers with the difficult task of balancing between the sometimes conflicting preferences of young cancer patients and their parents. This requires an ongoing evaluation of patients' and parents' needs and preferences at different stages of the illness. The use of a screening tool for evaluating patients' and parents' communication needs and preferences in daily practice may be useful in this respect. With the use of the data provided by our online focus groups, we are currently developing such tools.

## Competing interests

The author(s) declare that they have no competing interests.

## Authors' contributions

MZ and KT developed the content of the focus group websites, functioned as moderators in the online focus groups, and selected key aspects of focus group contributions. MZ drafted the manuscript. KT and JB designed the study and acquired funding. KT, SvD, PH, WK and JB have been involved in critically revising the manuscript. All authors have read and approved the final manuscript.

## Pre-publication history

The pre-publication history for this paper can be accessed here:


